# Development of a Culturally Sensitive Intervention for Cervical Cancer Screening Promotion for Latinx Transgender Individuals

**DOI:** 10.1002/cam4.71494

**Published:** 2026-01-11

**Authors:** Alíxida Ramos‐Pibernus, Mario Bermonti‐Pérez, David Mejías‐Serrano, Fabián Moreta‐Ávila, Paola Carminelli‐Corretjer, Nelmit Tollinchi‐Natali, Malynie Blanco, Lellanes Justiz, Marta Febo, Matthew B. Schabath, Ash B. Alpert, Eliut Rivera‐Segarra

**Affiliations:** ^1^ School of Behavioral and Brain Sciences Ponce Health Sciences University Ponce Puerto Rico; ^2^ Ponce Research Institute Ponce Health Sciences University Ponce Puerto Rico; ^3^ Independent Community Researcher San Francisco California USA; ^4^ School of Dental Medicine Ponce Health Sciences University Ponce Puerto Rico; ^5^ School of Medicine Ponce Health Sciences University Ponce Puerto Rico; ^6^ H. Lee Moffitt Cancer Center & Research Institute Tampa Florida USA; ^7^ Yale Cancer Center New Haven Connecticut USA

**Keywords:** cervical cancer prevention, intervention, standardize patient simulation, transmasculine

## Abstract

**Introduction:**

Trans men and non‐binary people face some of the most challenging cancer health disparities. Primary care physicians could play a key in addressing these, but many clinicians' lack the necessary skill to discuss cervical screening with trans people as these are not routinely taught in medical school. Thus, the objective of this study was to develop an intervention to foster medical students' skills for cervical cancer screening and to examine its initial impact and feasibility.

**Methods:**

Our research team is comprised of academic researchers, clinicians, and community members. Together, we developed a 2‐h intervention which we implemented using Standardized Patient Simulations (TM actors portraying the role of a TM patient) to observe provider behaviors (general care behaviors, gender affirming behaviors and cervical cancer preventive behaviors) and self‐reported measures to examine study outcomes. The total sample consisted of 37 third‐year medical students. Welch's *t*‐test was used to compare the intervention effects on all outcomes.

**Results:**

Results suggest the intervention had medium to large effects on all examined behaviors. Behaviors improved in the experimental group compared to the control group and all changes were statistically significant. In general, the intervention was seen as feasible and appropriate with participants mentioning it was “very helpful” and emphasizing the importance of discussing trans health care as part of their medical training as this improves their “confidence.”

**Discussion:**

Although the sample size was small, results show a potentially promising intervention. We provide an overview of the content of the intervention and discuss future research directions.

## 1 Introduction

Transmen (TM; individuals who have gender identities as men and who were assigned female at birth) and non‐binary individuals (NB; individuals assigned a female gender based on their sex at birth who identify as a man, male, or another diverse non‐binary gender identity) face some of the most challenging cancer health disparities [1]. TM and NB individuals are at a higher risk of screening‐detectable cancers, such as cervical cancer (CC) [2, 3, 4]. Latinxs (we use this gender expansive term and to be inclusive of varied gender identities and to circumvent the default use of masculine terminology) [5, 6, 7] are a particularly at‐risk population, as they have the highest incidence of CC among all ethnic groups in the United States [8, 9]. Thus, Latinx TM‐NB (LTM‐NB) who face intersectional oppression due to racism and transphobia face an even higher CC risk [10].

Primary care physicians are key in addressing disparities in cervical cancer screening and incidence through their patient‐provider clinical interactions with LTM‐NB individuals [11, 12, 13, 14, 15, 16]. Unfortunately, factors such as the gendered nature of screening testing, “misgendering” or use of incorrect pronouns, name or gender in clinical interactions, and TM‐NB avoidance of discussing body parts associated with their assigned at birth gender continue to characterize most patient‐provider interactions with LTM‐NB individuals [2, 17, 18, 19]. In addition, providers receive little training during and after medical school and may have stigmatizing attitudes and behaviors towards LTM‐NB people, which further hamper CC prevention and screening efforts in primary care [20, 21, 22].

Despite the broad availability of LGBT guidelines to foster cancer prevention among TM‐NB individuals [23, 24, 25], several barriers to successful cancer screening programs for transgender people warrant further attention. Some of these gaps include: (1) exclusive reliance on the use of self‐report measures to examine intervention outcomes, (2) lack of theory‐driven interventions to address the intersectional nature of multiple forms of oppression, such as those experienced by LTM‐NB communities, and (3) absence of evidence of feasibility, acceptability, and appropriateness of particular interventions [25, 26, 27]. We, therefore, sought to address these gaps by developing an intervention to increase medical students' cervical cancer screening behaviors in clinical interactions with LTM‐NB individuals and examining the intervention's feasibility, acceptability, and appropriateness.

**FIGURE 1:**
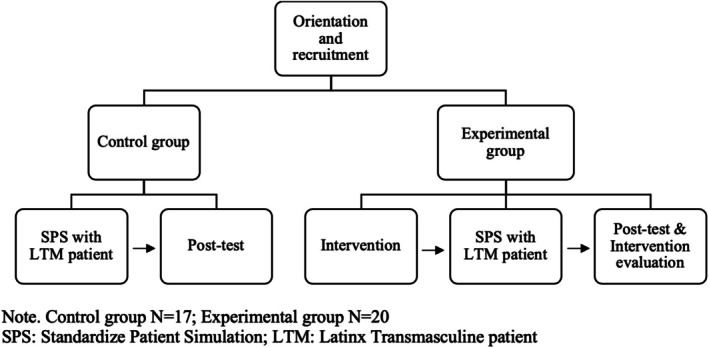
Study procedures. Control group *N* = 17; experimental group *N* = 20. SPS, standardized patient simulations; LTM, Latin transmasculine patient.

## 2 Methods

### 2.1 Design and Procedures

This study is part of a project examining barriers and facilitators for LTM individuals' cervical cancer screening. The research was approved by the Ponce Health Sciences University Institutional Review Board (IRB# 1903007737). Our research methodology employs community‐based participatory research, and our team includes academic researchers, clinicians, and community members. Together, we co‐developed the intervention for cervical cancer screening promotion for LTM‐NB individuals. Our intervention used Standardized Patient Simulations (SPS), which consisted of clinical interactions with actors portraying the role of a patient. Such simulations are routinely used in medical schools to examine medical students' competencies and observable behaviors [28, 29]. The data collected included self‐reported measures of intervention acceptability, feasibility and appropriateness, and observational data from the Cervical Cancer Preventive Behaviors Inventory (CCPBI) (Figure 1).

### 2.2 Participants

The total sample consisted of 37 third‐year medical students, all of whom were scheduled to participate in their third year monthly SPS rotations. Participants were recruited based on convenience sampling through the SPS program staff. Inclusion criteria were (1) 21 years of age or older and (2) in their third year of medical school. Interested students were provided with an orientation including an overview of the study and details about risks associated, and if they chose to participate, completed a consent form and sociodemographic questionnaire. This study did not involve randomization and masking. The first group of participants was assigned to the control condition (*n* = 17), while the second group was assigned to the experimental (*n* = 20). Subsequent to this, they engaged in an SPS simulation with a LTM patient.

### 2.3 Intervention: CC‐TRAINED—Cervical Cancer Screening Promotion for LTM‐NB Individuals

The research team developed a brief intervention to improve medical students' competencies for cervical cancer screening promotion for LTM‐NB individuals. To inform our intervention, we used the Gender Affirming Framework, which posits that social recognition and support for trans gender identity and expression in high‐risk contexts (e.g., patient/provider interactions) is key to reducing health disparities [11, 30]. In addition, we used the application of Intersectionality Theory to clinical medicine, in other words the recognition that intersecting forms of oppression impact patient‐clinician interactions and must be understood and intervened upon to address health disparities [31]. We used Social Cognitive Theory to develop our conceptual model of the intervention's mechanisms of change (i.e., through knowledge, attitudes, and behaviors) [32]. Based on our theoretical frameworks, we aimed to increase medical students' competencies for cervical cancer screening promotion for LTM individuals by: (1) increasing knowledge about LTM healthcare needs, (2) reducing negative attitudes towards LTM, and (3) increasing behavioral skills for the provision of care to LTM individuals.

The format of the intervention consisted of a face‐to‐face 2‐h lecture with a PowerPoint presentation including information about (1) cervical cancer disparities among LTM‐NB individuals; (2) intersectionality and cultural humility; (3) gender‐affirming cervical cancer prevention; and (4) steps for effective clinical interactions with LTM‐NB individuals. At the end of the lecture, participants engaged in a 30‐min role‐play and received feedback during which the participants were able to ask questions. The intervention was facilitated by the first author who is a licensed clinical psychologist with experience providing clinician training on gender‐affirming practices. Other members of the team were present during the intervention and provided input during role‐plays. After participating in the intervention, participants continued with their SPS, which included our LTM case.

### 2.4 Standardized Patient Simulation

The SPS case script, jointly developed by the research team, focused on an LTM individual who presented with pelvic pain and irregular bleeding. A LTM actor trained by the SPS staff in the study's script performed all cases. This LTM SPS was interspersed among their other scheduled SPS cases. All participants were aware they would engage in an observed SPS with a LTM patient at some point during their SPS rotations. However, they were unaware of the case specifics or its scheduling. All SPS interactions were video recorded. During the SPS interaction, the actor: (1) described their symptoms, (2) reported a double mastectomy (if prompted), (3) disclosed gender identity (if prompted), (4) reported current testosterone use (if prompted), (5) reported a history of cervical cancer in the family (if prompted), and (6) reported no previous history of HPV vaccination or screening (if prompted). These interactions lasted for approximately 20 min in which medical students had to: (1) obtain the patient's medical history, (2) discuss identified symptoms and risks, and (3) recommend treatment and testing.

### 2.5 Measures

#### 2.5.1 Sociodemographic Questionnaire

The team developed a self‐report questionnaire to collect participants' demographics including age, gender, sexual orientation, income, religion, social class, contact with LTM, current year of medical training, among other covariates.

#### 2.5.2 Cervical Cancer Preventive Behaviors Inventory (CCPBI)

This observational inventory was developed by the research team based on prior research [33, 34, 35], establishing observational measures for SPS with the input of LTM participants, physicians, and medical students. The CCPBI assesses 38 nonverbal and verbal behaviors including those relevant to any interaction with a patient (general behaviors) and behaviors applicable to LTM‐NB (gender‐affirming behaviors and cervical cancer preventive behaviors). A trained observer assessed all behaviors using a three‐point scale with the following values: manifested, unsure, and not manifested.

#### 2.5.3 Intervention Acceptability Questionnaire

This questionnaire was developed by the research team to measure how participants (medical students) perceived the intervention. It is composed of 4 open‐ended questions and 10 items answered in a 5‐point Likert format.

#### 2.5.4 Acceptability of Intervention Measure

This 4‐item measure, answered in a 5‐point Likert format, examines participant satisfaction about the intervention. It has a high reliability (alpha 0.85) [36].

#### 2.5.5 Implementation Appropriateness Measure

This 4‐item measure, answered in a 5‐point Likert format, examines perceived fit and relevance of the intervention. It has excellent reliability (alpha 0.91) [36].

#### 2.5.6 Feasibility of Intervention Measure

This 4‐item measure, answered in a 5‐point Likert format, examines participants' perception about the potentially successful implementation of the intervention. It has a good reliability (alpha 0.89) [36].

### 2.6 Data Analysis

Quantitative data were analyzed using IBM SPSS Statistics Version 27 (Armonk, New York) unless otherwise specified. Three intervention outcomes were derived from the CCPBI by aggregating the items for its three subscales: general behaviors, gender affirming behaviors, and cervical cancer preventive behaviors. Means with their 95% CI and SD were used to describe the data for the intervention and control group. Welch's *t*‐test were used to compare the intervention effects on all outcomes with α set to 0.05 and two‐tailed hypothesis tests. Hedges' G was calculated using Python's Pingouin package [37, 38, 39, 40, 41, 42] to estimate the standardized effect size. The False Discovery Rate was controlled with the Benjamini/Hochberg procedure [37] using Python's statsmodels package. For inferential analyses, *t*, *p*, 95% CI for mean difference and effect sizes are reported.

Qualitative data was analyzed using a rapid thematic analysis – an innovative technique to obtain targeted qualitative data in a shorter period of time [43]. Following the rapid analysis process recommendations, the team developed a summary table with responses to open‐ended questions.

## 3 Results

### 3.1 Quantitative Results

Sociodemographic data showed that half of participants (54%) selected female assigned at birth, 89% identified as heterosexual, and most of the sample (83.3%) had not had any training on transgender health issues. Table 1 includes a more detailed description of participants' sociodemographic characteristics.

**TABLE 1:** Sample characteristics.

Variable	Frequency	Frequency (%)
Assigned at birth sex		
Male	17	45.9
Female	20	54.1
Sexual orientation		
Heterosexual	33	89.2
Homosexual/Lesbian	2	5.4
Bisexual	2	5.4
Home area		
Urban	36	97.3
Rural	1	2.7
Marital status		
Single	25	67.6
Married	7	18.9
I live with my partner (not legally married)	5	13.5
Religious group		
Catholics	14	37.8
Protestants	6	16.2
None	13	35.1
Other	4	10.8
Income		
Less than $50,000	19	51.4
From $50,001 to $60,000	2	5.4
From $60,001 to $70,000	4	10.8
From $70,001 to $80,000	1	2.7
From $80,001 to $90,000	1	2.7
From $90,001 to $100,000	3	8.1
More than $100,000	7	18.9
Training, seminar, or workshop related to transgender health issues		
Yes	6	16.7
No	30	83.3
Training on social/psychological aspects of transgender health		
No	30	83.3
Yes	6	16.7
Training on body modifications and transgender health		
No	34	94.4
Yes	2	5.6
Completed hours of training		
0	24	80
1	1	3.3
4	4	13.3
5	1	3.3

*Note: N* = 37.

The intervention had medium to large effects on all outcomes, *g* > 0.50. The greatest improvement was in the general care behaviors, *g* = 1.143. The intervention effects on clinicians' behaviors related to gender and those related to cancer behaviors were very similar. All these improvements are statistically significant, *p* < 0.05, after controlling for the False Discovery Rate [38], but show uncertainty as seen in the lower bound of the confidence interval, which was sometimes smaller than one point on the appropriate scale. Table 2 presents the descriptive statistics for gender, cancer, and general care behaviors and the intervention effects on all outcomes.

**TABLE 2:** Descriptive statistics for control and intervention groups on gender, cancer, and general care behaviors and group comparisons.

Variable	Control			Intervention			Group comparison			
	Mean	SD	95% CI	Mean	SD	95% CI	*t*	*p* ^a^	Cohen's *d* ^b^	95% CI
Gender behaviors	15.40	3.07	[13.70, 17.10]	17.8	3.16	[16.32, 19.28]	2.263	0.043	0.735	[0.24, 4.56]
Cancer behaviors	13.47	4.24	[11.12, 15.81]	18.1	3.74	[16.35, 19.85]	3.363	0.006	0.752	[1.81, 7.46]
General care behaviors	44.53	7.99	[40.11, 48.96]	49.85	6.30	[16.35, 19.85]	2.218	0.043	1.43	[0.18, 10.45]

*Note: N*_control group_ = 15. *N*_intervention group_ = 20.

^a^ Corrected *p*‐values using Benjamini/Hochberg procedure for controlling the False discovery rate.

^b^ The Hedges *g* effect size estimator was used.

### 3.2 Implementation Assessment Results

Results for intervention's acceptability (Tables 3 and 4) showed that most participants felt the intervention was satisfactory in nearly all the domains evaluated. Participants were less agreeable with the intervention format and how competent they felt to provide care to transgender patients. Most of the participants rated the intervention as acceptable. In terms of the intervention appropriateness (Table 5), participants agreed that the intervention was adequate in all domains measured with a mean between 4.55 and 4.45 for all items. Finally, the intervention was evaluated as feasible by participants with a mean fluctuating from 4.55 to 4.65 in all items (Table 6).

**TABLE 3:** Intervention acceptability questionnaire (IAQ).

Variables	Minimum	Maximum	Mean	SD	95% CI
IAQ_total	37	49	45.3	3.57	[43.63, 46.97]
Facilitators were knowledgeable about the transgender men and cervical cancer topic	4	5	4.85	0.37	[4.68, 5.02]
Facilitators responded well to questions from the audience	4	5	4.8	0.41	[4.61, 4.99]
The lecture was an adequate format to deliver intervention content	4	5	4.8	0.41	[4.61, 4.99]
The lecture format was useful to increase my knowledge on how to provide cancer preventive care to Latinx transmen	4	5	4.75	0.44	[4.54, 4.96]
The lecture format was useful to increase my skills to provide cancer preventive care to Latinx transmen	4	5	4.8	0.41	[4.61, 4.99]
The role play was useful to improve my skills to provide cancer preventive care to Latinx transmen	3	5	4.75	0.55	[4.49, 5.01]
The use of another format (i.e., online video, getting to know a transman) would have been more useful to improve my knowledge and skills to provide cancer preventive care to Latinx transmen	1	5	3.75	1.45	[3.07, 4.43]
The material covered was important to understand how to provide cancer preventive care to Latinx transmen	4	5	4.8	0.41	[4.61, 4.99]
I would recommend this intervention to other medical students and physicians	4	5	4.85	0.37	[4.68, 5.02]
How successful has this intervention been in helping you to feel competent to provide cancer preventive care to Latinx transmen?	2	5	3.15	0.75	[2.8, 3.5]

*Note: N* = 20.

Abbreviations: CI = confidence intervals, SD = standard deviation.

**TABLE 4:** Acceptability of intervention measure.

Variables	Minimum	Maximum	Mean	SD	95% CI
Acceptability of intervention measure	14	20	18.35	2.13431	[17.35, 19.35]
This intervention meets my approval	4	5	4.6	0.503	[4.36, 4.84]
This intervention is appealing to me	3	5	4.55	0.605	[4.27, 4.83]
I like the intervention	3	5	4.55	0.605	[4.27, 4.83]
I welcome the intervention	4	5	4.65	0.489	[4.42, 4.88]

*Note: N* = 20.

Abbreviations: CI = confidence intervals, SD = standard deviation.

**TABLE 5:** Intervention appropriateness measure.

Variables	Minimum	Maximum	Mean	SD	95% CI
Intervention appropriateness measure	12	20	18.9	2.19809	[17.87, 19.93]
The intervention seems fitting	3	5	4.55	0.605	[4.27, 4.83]
The intervention seems suitable	3	5	4.55	0.605	[4.27, 4.83]
The intervention seems applicable	2	5	4.45	0.759	[4.09, 4.81]
The intervention seems like a good match	3	5	4.55	0.605	[4.27, 4.83]

*Note: N* = 20.

Abbreviations: CI = confidence intervals, SD = standard deviation.

**TABLE 6:** Feasibility of the intervention measure.

Variables	Minimum	Maximum	Mean	SD	95% CI
Feasibility of intervention measure	12.00	20.00	18.05	2.42	[16.92, 19.18]
The intervention seems implementable	3.00	5.00	4.60	0.60	[4.32, 4.88]
The intervention seems possible	3.00	5.00	4.65	0.59	[4.38, 4.92]
The intervention seems doable	3.00	5.00	4.65	0.59	[4.38, 4.92]
The intervention seems easy to use	3.00	5.00	4.55	0.76	[4.19, 4.91]

*Note: N* = 20.

Abbreviations: CI = confidence intervals, SD = standard deviation.

### 3.3 Qualitative Results

Most participants provided answers to the open‐ended questions in the Intervention Acceptability Questionnaire. In general, participants recognized that the intervention was “very helpful” and an “integral part of the learning process”. Participants recognized the importance of discussing trans health care as part of their training as this can improve their “confidence” to ask appropriate questions. Finally, they provided several recommendations to improve the intervention. Specifically, regarding the need to include the intervention earlier during their training, having more “first‐hand experiences” and receiving feedback after the SPS. Another important recommendation to improve the intervention was regarding the format or delivery methods: “use of audio visuals, videoclips of intervention.” Table 7 presents sample quotes from each of the questions.

**TABLE 7:** Responses to open ended questions of the IAQ.

Open ended questions	Sample narrative responses
Did you find the intervention to be helpful? If so, how?	Very helpful, this is one of those topics in which one doesn't really know how ignorant one is about the subject until actually having an open discussion This intervention is, in my opinion, an integral part of the learning process, since a good physician should be well prepared to provide their service to all patients seeking help Very helpful. I've been studying medicine for 3 1/2 years and this is the first clinical scenario with this patient population that I've had the chance to practice. We (students) are a lot more ignorant on the subject than what we realize
2 What were the most successful aspects of the intervention?	Explanation of terminologies. Statistics were provided. Presenters were non‐biased. Appropriate information well given To get rid of the shame of asking questions, like asking a transman if they still conserve their “female genitals” Helpful in making us aware of our language and medical management with transgender patients. It gives me more confidence when talking to these pts. respectfully
3 How can the intervention be improved?	I think the conference should be given to students before starting clinical rotations since it is full of very good general practices, not only regarding transgender population, but generally Just being able to experience it first‐hand. Had a couple of brick walls that were nice to face I would like feedback on the SP experience to see how I can improve and learn

*Note: N* = 20.

## 4 Discussion

In order to address the health disparities experienced by LTM, medical school curriculums need to integrate approaches that combine knowledge acquisition and attitude change (i.e., guidelines, post‐intervention surveys) with skill development strategies that can be transferable to patient‐provider interactions [44]. This is one of the first studies to date examining the preliminary impact of a culturally tailored intervention to improve gender‐affirming cervical cancer screening behaviors among medical students in clinical interactions with LTM patients.

One of the main findings of this study suggests that the intervention can improve medical students' actual behaviors in clinical interactions with LTM (i.e., general, gender‐affirming, and cervical cancer prevention behaviors). This is an important finding that provides empirical evidence to what current literature has suggested regarding the importance of focusing on behavior change and skills development, particularly when working with socially stigmatized populations [35, 45, 46, 47]. It also aligns with and expands on what different medical organizations, institutions, and community partners have highlighted regarding the need for LGBT health education for health professionals and the importance of embedding it into medical school's curriculum [48, 49]. However, with some exceptions [50, 51], most medical schools' curriculums are usually limited to introductory topics and definitions for gender and sexual diversity [52, 53]. As such, information about gender‐affirming care or specific health considerations for LTM individuals such as cancer prevention is usually limited, which often leads medical professionals to feel inadequate and with limited skills when providing health services to LTM [20]. Moreover, it increases health inequities for LTM individuals who often receive substandard or denial of health care. The findings from this study regarding feasibility, acceptability, and appropriateness could suggest the intervention's potential for integration into the medical curriculum. Nonetheless, participants also suggested that the intervention delivery format could be further improved by including additional visuals or video clips and receiving feedback after the SPS interaction, instead of after the lecture's role‐play. Integrating these recommendations will be crucial to sustain the intervention within the medical school curriculum in the long term.

Our study has a number of strengths. Drawing from current research evidence on community participation in medical training [52] and the epistemic peerhood model proposed by Weingartner et al. [54], which states that “broad community input about best practices for representing gender diversity in patient simulation,” the team co‐developed the study design and SPS case with transmasculine people. In this study, it was not community input alone as suggested by the literature, but rather integration and collaboration which foster engagement of people with lived experiences in study design and the education of medical students. An additional important feature of this study is that the actual SPS case was performed by a transmasculine actor who portrayed the role of a patient, providing study participants with professional contact and an accurate depiction of transmasculine people. Previous research has documented that interactions with LTM are key to modifying attitudes and behaviors among health professionals [45].

This study has also some limitations. Firstly, the small sample size may limit the generalizability of the findings and the potential to fully examine the intervention's effect on study outcomes. Secondly, the focus on one medical institution alone limits the sample and understanding of potential feasibility, acceptability, and appropriateness challenges at other sites and populations, which is key for scaling up efforts in the future. SPS being limited to one actor adds to the lack of exposure of medical professionals to the social barriers and limitations needed to address the healthcare needs of the LTM‐NB populations. Thirdly, the lack of randomization and blinding could be a potential source of bias. Finally, the pre‐post design and lack of follow‐up limit the understanding of the intervention's impact on behaviors over time. Despite these limitations, the focus on the development of a theoretically driven intervention aiming at improving medical students' skills to address cervical cancer prevention for LTM‐NB represents an important first step and model to address cancer disparities among LTM‐NB.

## Author Contributions

Alíxida Ramos‐Pibernus contributed to the conceptualization, supervision of data collection and analysis and drafted and review the initial version of the manuscript. Eliut Rivera‐Segarra, Matthew B. Schabath, and Ash B. Alpert contributed to the conceptualization and revision of the manuscript. Mario Bermonti‐Pérez conducted the quantitative data analysis and drafted the quantitative methods and results section. Paola Carminelli‐Corretjer and Nelmit Tollinchi‐Natali conducted the qualitative analysis and drafted the results. Fabián Moreta‐Ávila, David Mejías‐Serrano, Malynie Blanco, Lellanes Justiz, and Marta Febo contributed to data collection and revision of manuscript. All authors read and approved the final manuscript.

## Funding

This work was supported by the American Cancer Society under award IRG‐17‐173‐22. The content is solely the responsibility of the authors and does not necessarily represent the official views of the American Cancer Society or any other funding agency.

## Conflicts of Interest

The authors declare no conflicts of interest.

## Data Availability

As per our IRB protocol, the data used to develop this manuscript is not available. Any additional information can be requested by contacting the corresponding author Dr. Alixida Ramos‐Pibernus at aliramos@psm.edu.

## References

[1] A. R. Tabaac , M. E. Sutter , C. S. J. Wall , and K. E. Baker , “Gender Identity Disparities in Cancer Screening Behaviors,” American Journal of Preventive Medicine 54, no. 3 (2018): 385–393, 10.1016/j.amepre.2017.11.009. 29338956

[2] M. Agénor , J. M. White Hughto , S. M. Peitzmeier , et al., “Gender Identity Disparities in Pap Test Use in a Sample of Binary and Non‐Binary Transmasculine Adults,” Journal of General Internal Medicine 33, no. 7 (2018): 1015–1017, 10.1007/s11606-018-4400-3. 29654599 PMC6025652

[3] J. Potter , S. M. Peitzmeier , I. Bernstein , et al., “Cervical Cancer Screening for Patients on the Female‐To‐Male Spectrum: A Narrative Review and Guide for Clinicians,” Journal of General Internal Medicine 30, no. 12 (2015): 1857–1864, 10.1007/s11606-015-3462-8. 26160483 PMC4636588

[4] S. M. Peitzmeier , M. Agénor , I. M. Bernstein , et al., ““It Can Promote an Existential Crisis”: Factors Influencing Pap Test Acceptability and Utilization Among Transmasculine Individuals,” Qualitative Health Research 27, no. 14 (2017): 2138–2149, 10.1177/1049732317725513. 28836483

[5] L. N. Borrell and S. E. Echeverria , “The Use of Latinx in Public Health Research When Referencing Hispanic or Latino Populations,” Social Science & Medicine 302 (2022): 114977, 10.1016/j.socscimed.2022.114977. 35504084

[6] A. R. Miranda , A. Perez‐Brumer , and B. M. Charlton , “Latino? Latinx? Latine? A Call for Inclusive Categories in Epidemiologic Research,” American Journal of Epidemiology 192, no. 12 (2023): 1929–1932, 10.1093/aje/kwad149. 37392097 PMC10988219

[7] S. R. Ramos , C. J. Portillo , C. Rodriguez , and J. I. Gutierrez , “Latinx: Sí, se Puede? A Reflection on the Terms Past, Present, and Future,” Journal of Urban Health 100, no. 1 (2023): 4–6, 10.1007/s11524-022-00690-y. 36260246 PMC9918645

[8] American Cancer Society , Cancer Facts & Figures for Hispanics/Latinos 2015–2017 (American Cancer Society, 2015).

[9] American Cancer Society , “Cervical Cancer Incidence Rates Remain Higher in Hispanic/Latina Women,” American Cancer Society Action Network, Inc, 2017, acscan.org.

[10] National Institute of Minority and Health Disparities , “Sexual and Gender Minorities Formally Designated as a Health Disparity Population for Research Purposes,” Directors Message, 2016.

[11] Center of Excellence for Transgender Health , Guidelines for the Primary and Gender‐Affirming Care of Transgender and Gender Nonbinary People (Center of Excellence for Transgender Health, University of California San Francisco, 2016), 199.

[12] K. L. Eckstrand and J. Ehrenfeld , Lesbian, Gay, Bisexual, and Transgender Healthcare: A Clinical Guide to Preventive, Primary, and Specialist Care (Springer International Publishing, 2017), 10.1080/00918369.2017.1321392.

[13] M. Agénor , S. M. Peitzmeier , I. M. Bernstein , et al., “Perceptions of Cervical Cancer Risk and Screening Among Transmasculine Individuals: Patient and Provider Perspectives,” Culture, Health & Sexuality 4 (2016): 1–15, 10.1080/13691058.2016.1177203. 27142466

[14] S. J. Curry , A. H. Krist , D. K. Owens , et al., “Screening for Cervical Cancer,” JAMA 320, no. 7 (2018): 674, 10.1001/jama.2018.10897. 30140884

[15] K. Nessler , S. K. F. Chan , F. Ball , et al., “Impact of Family Physicians on Cervical Cancer Screening: Cross‐Sectional Questionnaire‐Based Survey in a Region of Southern Poland,” BMJ Open 9, no. 8 (2019): e031317, 10.1136/bmjopen-2019-031317. PMC6720140 31473624

[16] S. Stumbar , “The Responsibility of Family Physicians to Our Transgender Patients,” American Family Physician 98, no. 11 (2018): 635. 30485051

[17] S. M. Peitzmeier , S. L. Reisner , P. Harigopal , and J. Potter , “Female‐To‐Male Patients Have High Prevalence of Unsatisfactory Paps Compared to Non‐Transgender Females: Implications for Cervical Cancer Screening,” Journal of General Internal Medicine 29, no. 5 (2014): 778–784, 10.1007/s11606-013-2753-1. 24424775 PMC4000345

[18] M. McDowell , D. J. Pardee , S. Peitzmeier , et al., “Cervical Cancer Screening Preferences Among Trans‐Masculine Individuals: Patient‐Collected Human Papillomavirus Vaginal Swabs Versus Provider‐Administered Pap Tests,” LGBT Health 4, no. 4 (2017): 252–259, 10.1089/lgbt.2016.0187. 28665783

[19] S. L. Reisner , M. B. Deutsch , S. M. Peitzmeier , et al., “Comparing Self‐ and Provider‐Collected Swabbing for HPV DNA Testing in Female‐To‐Male Transgender Adult Patients: A Mixed‐Methods Biobehavioral Study Protocol,” BMC Infectious Diseases 17, no. 1 (2017): 444, 10.1186/s12879-017-2539-x. 28645254 PMC5481878

[20] S. L. R. Madera , N. V. Díaz , M. Padilla , et al., ““Just Like Any Other Patient”: Transgender Stigma Among Physicians in Puerto Rico,” Journal of Health Care for the Poor and Underserved 30, no. 4 (2019): 1518–1542, 10.1353/hpu.2019.0089. 31680112 PMC7233420

[21] A. G. Ramos‐Pibernus , E. R. Rivera‐Segarra , S. L. Rodríguez‐Madera , N. Varas‐Díaz , and M. Padilla , “Stigmatizing Experiences of Trans Men in Puerto Rico: Implications for Health,” Transgender Health 5, no. 4 (2020): 234–240, 10.1089/trgh.2020.0021. 33381650 PMC7759285

[22] K. Kosenko , L. Rintamaki , S. Raney , and K. Maness , “Transgender Patient Perceptions of Stigma in Health Care Contexts,” Medical Care 51, no. 9 (2013): 819–822, 10.1097/MLR.0b013e31829fa90d. 23929399

[23] I. Bernstein , S. Peitzmeier , J. Potter , and S. L. Reisner , If You Have It, Check It: Overcoming Barriers to Cervical Cancer Screening With Patients on the Female‐To‐Male Transgender Spectrum (National LGBT Health Education Center, 2015).

[24] R. Rollston , Promoting Cervical Cancer Screening Among Female‐To‐Male Transmasculine Patients (Fenway Institute, 2019), 22.

[25] C. L. Fosmore , S. Sullivan , A. F. Brouwer , et al., “Strategies to Optimize Cervical Cancer Screening Rates Among Transgender and Gender‐Diverse People Assigned Female at Birth,” Journal of General Internal Medicine 39, no. 16 (2024): 3333–3338, 10.1007/s11606-024-09026-9. 39313668 PMC11618264

[26] M. R. Brottman , D. M. Char , R. A. Hattori , R. Heeb , and S. D. Taff , “Toward Cultural Competency in Health Care,” Academic Medicine 95, no. 5 (2020): 803–813, 10.1097/ACM.0000000000002995. 31567169

[27] M. Kano , N. Sanchez , I. Tamí‐Maury , B. Solder , G. Watt , and S. Chang , “Addressing Cancer Disparities in SGM Populations: Recommendations for a National Action Plan to Increase SGM Health Equity Through Researcher and Provider Training and Education,” Journal of Cancer Education 35, no. 1 (2018): 44–53, 10.1007/s13187-018-1438-1. PMC10368403 30377952

[28] D. Oller , “Cancer Screening for Transgender Patients: An Online Case‐Based Module,” MedEdPORTAL: Journal of Teaching and Learning Resources 15 (2019): 10796, 10.15766/mep_2374-8265.10796. PMC6376892 30800996

[29] C. A. Bohnert , R. M. Combs , E. J. Noonan , A. E. Weathers , and L. A. Weingartner , “Gender Minorities in Simulation: A Mixed Methods Study of Medical School Standardized Patient Programs in the United States and Canada,” Simulation in Healthcare: The Journal of the Society for Simulation in Healthcare 16, no. 6 (2021): e151–e158, 10.1097/SIH.0000000000000532. 33273422

[30] D. M. Gates , E. Fitzwater , and S. Telintelo , “Using Simulations and Standardized Patients in Intervention Research,” Clinical Nursing Research 10, no. 4 (2001): 387–400, 10.1177/10547730122159012. 11881950

[31] J. Sevelius , “Gender Affirmation: A Framework for Conceptualizing Risk Behavior Among Transgender Women of Color,” Sex Roles 68, no. 11–12 (2013): 675–689, 10.1016/j.surg.2006.10.010.Use. 23729971 PMC3667985

[32] Y. Wilson , A. White , A. Jefferson , and M. Danis , “Intersectionality in Clinical Medicine: The Need for a Conceptual Framework,” American Journal of Bioethics 19, no. 2 (2019): 8–19, 10.1080/15265161.2018.1557275. 30784384

[33] A. Bandura , “Social Cognitive Theory of Personality,” in Handbook of Personality, ed. L. Pervin and O. John (Guilford Publications, 1999), 154–196.

[34] E. Rivera‐Segarra , A. Ramos‐Pibernus , M. Blanco , L. Justiz , D. Mejías , and J. Silva , “Standardized Patients to Address Transmasculine Health Disparities,” In: RCMI National Conference‐Collaborative Solutions to Improve Minority Health & Reduce Health Disparities 2019.

[35] A. Ramos‐Pibernus , P. Carminelli‐Corretjer , M. Bermonti‐Pérez , et al., “Examining Cervical Cancer Preventive Behaviors for Latinx Transmasculine Individuals Among Medical Students,” International Journal of Environmental Research and Public Health 18, no. 3 (2021): 851, 10.3390/ijerph18030851. 33498187 PMC7863948

[36] E. Rivera‐Segarra , A. Ramos‐Pibernus , M. Blanco , P. Carminelli‐Corretjer , N. Tollinchi , and M. Bermonti‐Pérez , “Standardized Patients to Address Transmen Health Disparities [Abstract 29.08.003],” P R Health Sciences Journal 39, no. 1 (2020): 144.

[37] B. J. Weiner , C. C. Lewis , C. Stanick , et al., “Psychometric Assessment of Three Newly Developed Implementation Outcome Measures,” Implementation Science 12, no. 1 (2017): 108, 10.1186/s13012-017-0635-3. 28851459 PMC5576104

[38] Y. Benjamini and D. Yekutieli , “The Control of the False Discovery Rate in Multiple Testing Under Dependency,” Annals of Statistics 29, no. 4 (2001): 1165–1188, 10.1214/aos/1013699998.

[39] J. A. Durlak , “How to Select, Calculate, and Interpret Effect Sizes,” Journal of Pediatric Psychology 34, no. 9 (2009): 917–928, 10.1093/jpepsy/jsp004. 19223279

[40] S. Seabold and J. Perktold , “Statsmodels: Econometric and Statistical Modeling With Python,” 2010:92–96, 10.25080/Majora-92bf1922-011.

[41] R. Vallat , “Pingouin: Statistics in Python,” Journal of Open Source Software 3, no. 31 (2018): 1026, 10.21105/joss.01026.

[42] W. McKinney , “Pandas: A Foundational Python Library for Data Analysis and Statistics,” 2011.

[43] R. C. Gale , J. Wu , T. Erhardt , et al., “Comparison of Rapid vs In‐Depth Qualitative Analytic Methods From a Process Evaluation of Academic Detailing in the Veterans Health Administration,” Implementation Science 14, no. 1 (2019): 11, 10.1186/s13012-019-0853-y. 30709368 PMC6359833

[44] H. Moseson , N. Zazanis , E. Goldberg , et al., “The Imperative for Transgender and Gender Nonbinary Inclusion,” Obstetrics and Gynecology 135, no. 5 (2020): 1059–1068, 10.1097/AOG.0000000000003816. 32282602 PMC7170432

[45] J. A. Park and J. D. Safer , “Clinical Exposure to Transgender Medicine Improves Students' Preparedness Above Levels Seen With Didactic Teaching Alone: A Key Addition to the Boston University Model for Teaching Transgender Healthcare,” Transgender Health 3, no. 1 (2018): 10–16, 10.1089/trgh.2017.0047. 29344576 PMC5770129

[46] A. Marshall , S. Pickle , and S. Lawlis , “Transgender Medicine Curriculum: Integration Into an Organ System–Based Preclinical Program,” MedEdPORTAL Publications 13 (2017): 1–6, 10.15766/mep_2374-8265.10536. PMC6342291 30800738

[47] M. L. Pratt‐Chapman , K. Eckstrand , A. Robinson , et al., “Developing Standards for Cultural Competency Training for Health Care Providers to Care for Lesbian, Gay, Bisexual, Transgender, Queer, Intersex, and Asexual Persons: Consensus Recommendations From a National Panel,” LGBT Health 9, no. 5 (2022): 340–347, 10.1089/lgbt.2021.0464. 35443812 PMC9291720

[48] A. M. Pregnall , A. L. Churchwell , and J. M. Ehrenfeld , “A Call for LGBTQ Content in Graduate Medical Education Program Requirements,” Academic Medicine 96, no. 6 (2021): 828–835, 10.1097/ACM.0000000000003581. 34031304

[49] S. N. Dubin , I. T. Nolan , J. C. G. Streed , R. E. Greene , A. E. Radix , and S. D. Morrison , “Transgender Health Care: Improving Medical Students'; and Residents'; Training and Awareness,” Advances in Medical Education and Practice 9 (2018): 377–391, 10.2147/AMEP.S147183. 29849472 PMC5967378

[50] G. P. Quinn , J. Jimenez , C. D. Meade , et al., “Enhancing Oncology Health Care Provider's Sensitivity to Cultural Communication to Reduce Cancer Disparities: A Pilot Study,” Journal of Cancer Education 26, no. 2 (2011): 322–325, 10.1007/s13187-011-0223-1. 21479572 PMC3673723

[51] J. Seay , A. Hicks , M. J. Markham , et al., “Web‐Based LGBT Cultural Competency Training Intervention for Oncologists: Pilot Study Results,” Cancer 126, no. 1 (2020): 112–120, 10.1002/cncr.32491. 31524952

[52] E. J. Noonan , S. Sawning , R. Combs , et al., “Engaging the Transgender Community to Improve Medical Education and Prioritize Healthcare Initiatives,” Teaching and Learning in Medicine 30, no. 2 (2017): 1–14, 10.1080/10401334.2017.1365718. 29190167

[53] M. S. Minturn , E. I. Martinez , T. Le , et al., “Early Intervention for LGBTQ Health: A 10‐Hour Curriculum for Preclinical Health Professions Students,” MedEdPORTAL 17 (2021): 11072, 10.15766/mep_2374-8265.11072. 33473382 PMC7809930

[54] L. A. Weingartner , R. M. Combs , C. A. Bohnert , H. R. Decker , and E. J. Noonan , “Epistemic Peerhood as a Model to Improve Gender‐Affirming Care in Medical Education,” Teaching and Learning in Medicine 36, no. 1 (2022): 89–98, 10.1080/10401334.2022.2137169. 36314249

